# Experimental Study on Heat Conduction and Water Migration of Composite Bentonite Samples

**DOI:** 10.3390/ma17174211

**Published:** 2024-08-26

**Authors:** Gaosheng Yang, Bing Bai, Wenxuan Chen, Haitao Mao, Zhonghua Liu, Xiaoling Lan

**Affiliations:** 1 College of Urban and Rural Construction, Shanxi Agricultural University, Jinzhong 030801, China; yanggaosheng2022@163.com (G.Y.); 15700885247@163.com (W.C.); maohaitao1234@163.com (H.M.); lzh6175305@163.com (Z.L.); xiaolinglan@163.com (X.L.); 2 School of Civil Engineering, Beijing Jiaotong University, Beijing 100044, China

**Keywords:** GMZ bentonite, composite samples, joints, thermal conductivity, hydraulic conductivity

## Abstract

The joints of buffer material composite blocks as potential weak parts in the engineering barrier system of a high-level radioactive waste (HLW) repository must be studied in depth. Therefore, a laboratory experiment device suitable for unsaturated composite bentonite samples was developed. The evolution of temperature and volumetric water content at different locations of Gaomiaozi (GMZ) composite bentonite samples with time before and after simulated water inflow was measured by the experiment device. According to the experimental results, the thermal conductivity and hydraulic conductivity of the joint location after healing of the composite bentonite samples were obtained. The experimental results show that the change in the internal temperature of the composite bentonite samples is mainly affected by the temperature boundary and that the change in the internal water has little effect on it. In a short period of time, the loading of hydraulic boundary conditions only makes the volumetric water content of the soil near the hydraulic boundary increase significantly but has little effect on other locations. And, affected by the temperature boundary, the volumetric water content of the soil near the temperature boundary gradually decreases with time. The process of hydration swelling of the composite bentonite sample is accompanied by the adjustment of stress. The composite bentonite samples are continuously squeezed to the joint area after hydration swelling, the whole composite samples are generally homogenized, and the joints between the composite bentonite samples tend to heal. The thermal conductivity and permeability of the joint location after healing can meet the requirements of the engineering barrier of the HLW repository.

## 1. Introduction

The HLW deep geological repository uses a multi-barrier system that includes artificial barriers (waste-solidified body, packaging container, and buffer/backfill material) and natural barriers (surrounding rock). The surrounding and stacking high-pressure compacted bentonite blocks between the waste canister and geological body are one of the best choices for buffer materials in artificial barriers at present [1,2,3,4,5]. The main component of bentonite is montmorillonite, which is a layered clay mineral composed of two layers of silicon oxygen tetrahedron and one layer of aluminum oxygen octahedron. According to the types, contents, and crystallochemical properties of the exchangeable cations contained in montmorillonite, bentonite can be classified into sodium-based, calcium-based, magnesium-based, aluminum (hydrogen)-based, and other types of bentonite [6,7]. The preferred buffer/backfill material for China’s HLW repository is Inner Mongolia Gaomiaozi bentonite, which is a typical sodium-based bentonite. However, during the construction process, joints between the waste canister and bentonite block, between the surrounding rock and bentonite block, and between the bentonite blocks are inevitable. Moreover, due to the influence of radionuclide decay heat release and groundwater seepage, the buffer material is affected by multi-field coupling effects such as thermal-hydro-mechanical effects [8,9,10,11]. Although the high-pressure compacted bentonite block hydrates and swells under the action of groundwater and continuously squeezes toward the joint area, which makes the joint area gradually heal, for the overall performance of the buffer layer of the HLW repository, the joints area is still a potential weak link. Therefore, the performance of the buffer materials with joints under thermal-hydro-mechanical (THM) coupling conditions has become a key issue in the design and construction of HLW repository engineering barriers.

To study the influence of construction joints on the performance of buffer materials, domestic and foreign scholars have studied it from different perspectives in recent years. Popp et al. [12] studied the permeability experiment of a bentonite–sand mixture block with joints. The experimental results show that although there are joints in the sample, when it is completely saturated, the properties of the sample with joints are basically the same as those of the homogeneous complete sample, and the joints tend to heal completely. Hoffmann et al. [13] studied the hydration experiment of compacted bentonite granular materials. The experimental results show that the saturated permeability and swelling force of bentonite granular materials are mainly controlled by the total dry density of the sample, while the initial particle size distribution of bentonite particles has little effect on it. According to the experimental results, the relationship between the total dry density and the permeability coefficient of the sample is obtained. Chen et al. [14] carried out swelling force and permeability experiments on GMZ bentonite samples with different dry densities under different joint widths and carried out mercury porosity experiments on the hydrated samples. The results show that the existence of joints leads to a decrease in swelling force and an increase in the permeability of bentonite, thus reducing the self-sealing performance of bentonite in the repository. In the process of hydration, the existence of joints causes a change in pore size and quantity in bentonite, and the macroscopic performance shows an increase in the permeability of bentonite. Wang et al. [15] studied the permeability of bentonite samples with three kinds of joint materials. The experimental results show that the saturated permeability coefficient of samples sealed with different joint materials is between 5.15 × 10^−11^~7.16 × 10^−12^ m/s. The healing degree of the joint is proportional to the initial dry density of the sealing material, and the healing degree of the sample sealed with bentonite particles is the highest. Jia et al. [16] carried out an anisotropic swelling force experiment on GMZ bentonite samples with different dry densities under two kinds of joint widths. The experimental results show that the axial swelling force increases with the dry density faster than the radial swelling force increases with the dry density. At the same final dry density, the swelling force caused by the axial joint is smaller than that of the complete bentonite sample. Meng et al. [17] conducted a hydration experiment on GMZ bentonite samples with radial joints. The experimental results showed that the healing order of the joints from bottom to top was consistent with the hydration path. The healing of the joint was a gradually slowing process, which is shown in the semi-logarithmic coordinates as a linear relationship between the volume of the sample and the hydration time. However, the above studies can be summarized as the influence of joints on the performance of buffer materials under the coupling of hydro-mechanical fields, and the influence of radionuclide decay heat release on it is not considered. The change in temperature leads to the migration of water in the buffer material, which further affects the healing of the joints. Therefore, when studying the performance of buffer materials with joints, the temperature change cannot be ignored.

In this paper, a laboratory experiment device suitable for unsaturated composite bentonite samples was developed. The evolution of temperature and volumetric water content at different locations of GMZ composite bentonite samples with time before and after simulated water inflow was measured by the experiment device. According to the experimental results, the thermal conductivity and hydraulic conductivity of the joint location after healing of the composite bentonite samples were obtained. Finally, the healing process of the joint location of the composite bentonite sample was analyzed. The research results can provide a reference for the safety evaluation of the barrier system of the HLW repository project.

## 2. Experiment Device

**Figure 1:**
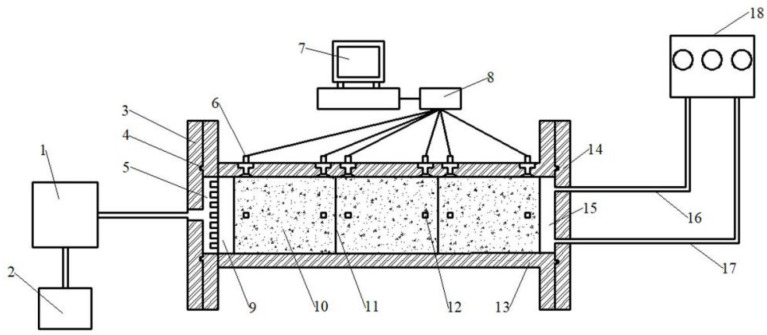
Diagram of the experiment device. Note: 1. Closed water tank; 2. high-pressure gas cylinder; 3. hydraulic boundary flange plate; 4. O-type rubber ring; 5. hydraulic boundary cavity; 6. sensor wire lead-out hole; 7. computer; 8. data acquisition instrument; 9. permeable stone; 10. bentonite samples; 11. joint location; 12. temperature and humidity sensor; 13. polypropylene cylinder; 14. temperature boundary flange plate; 15. temperature boundary cavity; 16. inlet pipe; 17. outlet pipe; 18. constant temperature water bath circulation device.

Figure 1 shows a schematic diagram of the developed experiment device for measuring the heat conduction and water migration parameters of unsaturated composite bentonite samples. The main body of the experiment device is made of adiabatic waterproof material (polypropylene material) into a cylindrical structure. The inner diameter of the cylinder is 10 cm, the wall thickness of the cylinder is 2 cm, and the length of the cylinder is 47 cm [8,18]. After the filling sample is completed, the two ends of the experiment device are connected with the stainless steel cavity (temperature and hydraulic boundary cavity) flange plate with movable bolts, and the O-type rubber ring is set for sealing. During the experiment, a high-temperature boundary is applied at the right end, which can be controlled at a higher constant temperature according to the experiment requirements. The high-temperature boundary is applied by a constant temperature water bath circulation device, which can provide a long-term higher constant temperature with an accuracy of ±0.1 °C. The hydraulic boundary is applied at the left end, and the closed water tank is pressurized by a high-pressure gas cylinder, and then the deionized water of the closed water tank is introduced into the hydraulic boundary cavity through the water pipe, so as to realize the long-term stable constant pressure water injection of the hydraulic boundary. In order to ensure that the deionized water in the hydraulic boundary cavity is uniformly infiltrated into the bentonite sample, a cylindrical permeable stone with a diameter of 10 cm and a thickness of 1 cm is set between the hydraulic boundary cavity and the bentonite sample. The temperature and humidity of the samples are collected by PT100 temperature sensor and EC-5 soil moisture sensor, and the corresponding acquisition instruments of temperature and humidity sensors are JY-DAM-TC16 and EM50, respectively. PT100 temperature sensor, EC-5 soil moisture sensor, JY-DAM-TC16, and EM50 are all produced at Huiye Industrial and Mining Machinery Equipment Company, Ltd. in Jining City, Shandong Province, China.

## 3. Experimental Scheme

The soil samples selected in this experiment were produced in Gaomiaozi Township, Inner Mongolia, China. The mineral composition is shown in Table 1. The basic physical property parameters are shown in Table 2. The GMZ bentonite was processed into a cylindrical sample with a dry density of 1.58 g/cm^3^ and a mass water content of 15%. The sample diameter was 100 mm, and the heights were 149 mm, 150 mm and 149 mm, respectively. The three bentonite samples were spliced in turn, the joints with a width of 1 mm were set at the splicing site, and the joints were filled with bentonite powder to form a composite sample with a total length of 450 mm and two joints in the middle.

**Figure 2:**
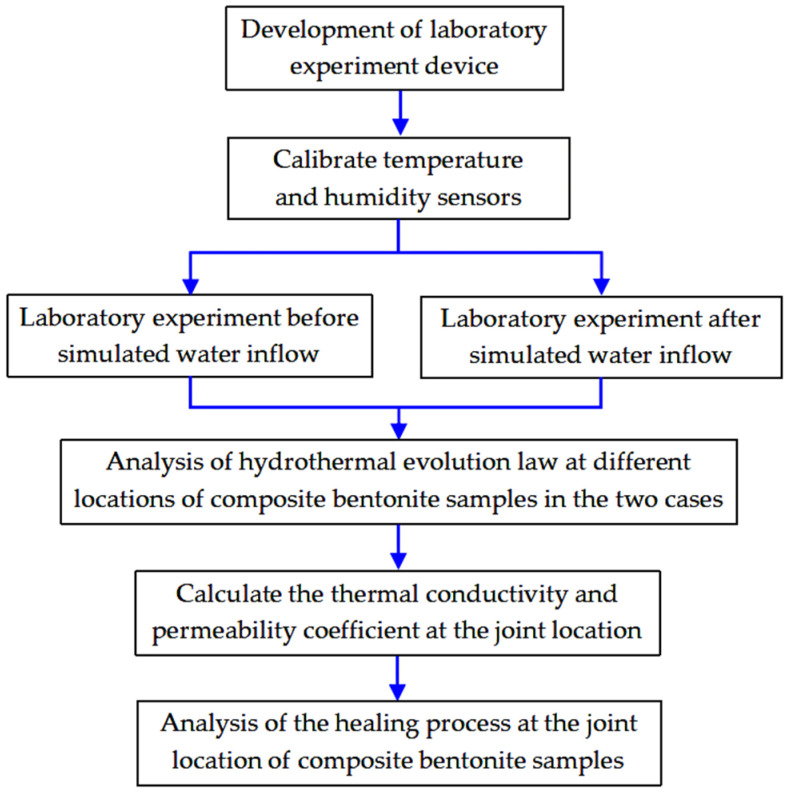
The experimental flow chart.

**Figure 3:**
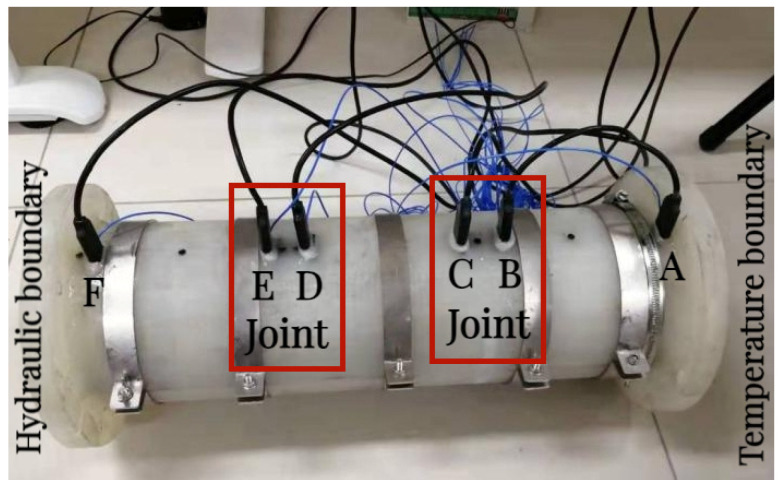
Main body of experiment device and sensor placement.

The experimental determination of heat conduction and the water migration law of composite bentonite samples includes two cases before and after simulated water inflow. For the case before simulated water inflow, we simulate the case before the external water flow into the repository. Both ends of the experiment device cylinder are set to impermeable boundary conditions. That is, the left and right ends of the experiment device cylinder are connected to the 25 °C and 75 °C impermeable heating cavities, respectively. At this time, under the action of boundary temperature load, the composite bentonite samples undergo heat conduction with the action of a temperature gradient and is accompanied by the water migration process inside the composite bentonite samples. For the case after simulated water inflow, we simulate the case after the external water flow into the repository. The impervious high-temperature boundary of 75 °C is set at the right end of the sample cylinder, the constant temperature permeable boundary of 25 °C is set at the left end, and the water pressure is set at 0.02 MPa (permeable holes and permeable stones are set on the bottom plate connected to the sample). At this time, under the action of boundary temperature load and hydraulic load, the composite bentonite samples produce thermal energy conduction and water flow penetration with the coupling effect of a temperature gradient and a hydraulic gradient. The experimental flow chart is shown in Figure 2. It is necessary to determine the corresponding heat conduction and hydraulic conductivity and the change process with time. The location of the temperature and humidity sensors is shown in Figure 3, and the distances from the temperature boundary are 15 mm (location A), 135 mm (location B), 165 mm (location C), 285 mm (location D), 315 mm (location E) and 435 mm (location F), respectively. During the experiment, the temperature of the laboratory is constant at 25 °C.

## 4. Experiment Results and Analysis

### 4.1. Temperature Evolution Law

**Figure 4:**
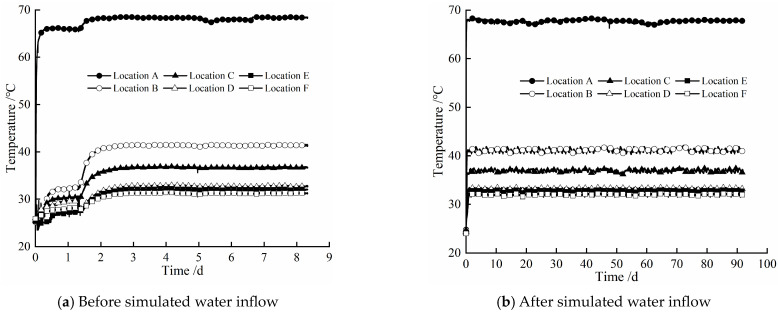
Variation in temperature at different locations of composite bentonite samples with time.

**Figure 5:**
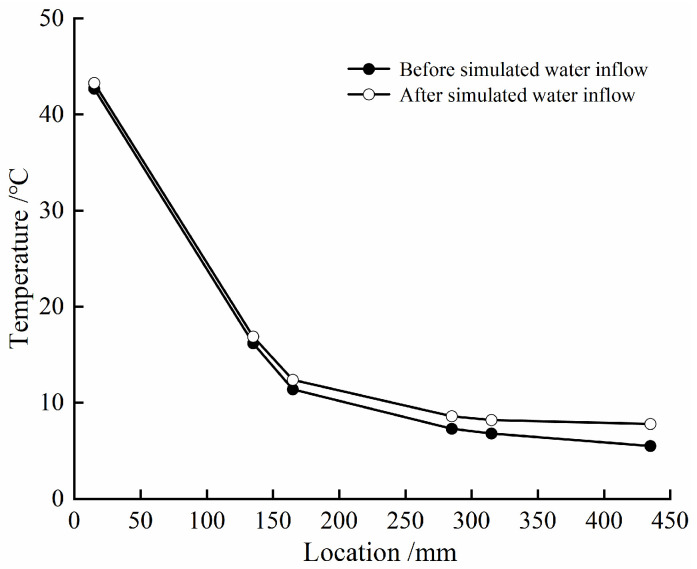
Temperature increase at different locations of composite bentonite samples.

Figure 4 gives the evolution law of temperature at different locations of the composite bentonite samples with time before and after simulated water inflow. According to Figure 4, it can be seen that the change trend of temperature at different locations of the composite bentonite samples with time is basically similar in the two cases. That is to say, whether the hydraulic boundary is set at the left end or not, due to the constant high-temperature boundary condition of 75 °C set at the right end of the experiment device cylinder, the temperature at different locations of the composite bentonite samples increases with different amplitudes, and the amplitude of temperature rise at different locations gradually decreases with the increase in distance from the high-temperature boundary at the right end. For these two cases, the amplitude of the temperature increase at different locations with the distance from the high-temperature boundary at the right end is shown in Figure 5. In the experiment before the simulated water inflow, when the experiment was carried out to 1.4 d, it can be found that the temperature at different locations of the composite bentonite samples increased significantly (Figure 4a). This is because during the experiment, although the main body of the experiment device was wrapped with a thermal insulation layer before the start of the experiment, it was found that there was still a large amount of heat dissipation. In order to minimize the heat dissipation during the experiment and reduce the impact on the experimental results, when the experiment was carried out to 1.4 d, the outer wall of the experiment device cylinder and the boundary flanges at both ends were further thermally insulated. Therefore, after 1.4 d, the temperature at different locations of the composite bentonite samples increased significantly.

Table 3 gives the initial temperature and final temperature of the experiment at different locations before and after simulated water inflow, where Δ*T*_1_ and Δ*T*_2_ are expressed as the difference between the initial temperature and final temperature at the same location in the two cases. By comparison, it can be found that the initial temperature at each location in the experiment after the simulated water inflow is slightly reduced. This is related to the side wall effect between the composite bentonite samples and the inner wall of the experiment device cylinder. That is, although before the start of the experiment, the gap between the composite bentonite samples and the inner wall of the experiment device cylinder was carefully filled with bentonite powder, due to the low permeability of the bentonite samples with higher dry density, under the action of water pressure, deionized water flowing from the left hydraulic boundary first flows into the gap between the composite bentonite samples and the inner wall of the experiment device cylinder and then fills the gap quickly, resulting in the initial temperature at different locations after simulated water inflow being slightly lower than that before simulated water inflow. With the hydration and swelling of bentonite powder filling in the gap between the composite bentonite samples and the inner wall of the experiment device cylinder, the seepage channel is gradually squeezed and closed. Under the continuous action of the hydraulic boundary at the left end, deionized water permeates along the pores of the bentonite sample towards the temperature boundary. The volumetric water content of the composite bentonite samples at different locations undergoes continuous changes, while the temperature change at the corresponding location is very small. This indicates that the change in the internal temperature of the composite bentonite samples is mainly affected by the high-temperature boundary at the right end, while the change in the internal water of the composite bentonite samples has little impact on it. Further comparing the final temperature of the experiment at different locations of the composite bentonite samples in the two cases, it can be found that the final temperature difference of the experiment at locations A and B near the high-temperature boundary at the right end is minor, while the final temperature of the experiment at locations E and F close to the hydraulic boundary at the left end is slightly higher than that before simulated water inflow (Table 3). This is mainly because the composite bentonite samples gradually hydrate under the action of the left hydraulic boundary. The volumetric water content of locations E and F increases significantly, while the volumetric water content of locations A and B does not change significantly, which makes the thermal conductivity of locations E and F increase after simulated water inflow, while the thermal conductivity of locations A and B is basically the same in the two cases [19,20,21]. Therefore, the final temperature difference of the experiment at locations A and B is small in these two cases, and the final temperature of the experiment at locations E and F after simulated water inflow is slightly higher than the final temperature of the experiment before simulated water inflow.

### 4.2. Water Evolution Law

**Figure 6:**
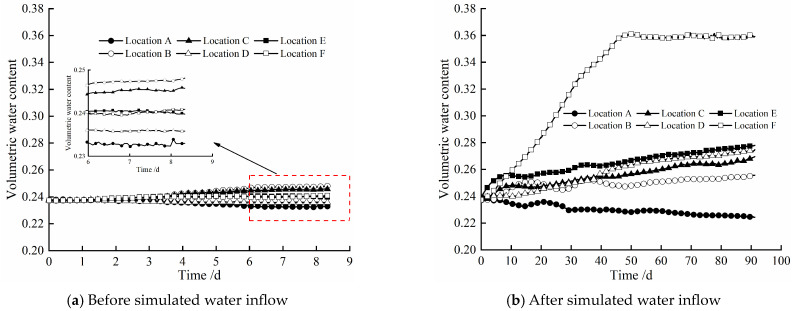
Variation in volumetric water content at different locations of composite bentonite samples with time.

Figure 6 shows the evolution law of volumetric water content at different locations of the composite bentonite samples with time before and after simulated water inflow. When dealing with the experimental results, the influence of the side wall effect between the bentonite samples and the inner wall of the experiment device cylinder was corrected. From Figure 6, it can be found that whether the hydraulic boundary is loaded at the left end of the experiment device cylinder has a significant effect on the volumetric water content of the composite bentonite samples near the hydraulic boundary. The experiment before the simulated water inflow, that is, when the hydraulic boundary is not set at the left end, due to the existence of the high-temperature boundary at the right end, the water inside the composite bentonite samples migrates from high temperature to low temperature under the action of the temperature gradient. The volumetric water content at the highest temperature location A is gradually decreased with time, while the volumetric water content at locations B and C, where the temperature is significantly lower than location A, is gradually increased with time. The volumetric water content at locations D, E, and F with a small temperature difference varies less with time (Figure 6a). The reason for the analysis is that the temperature difference at locations A, B, and C is larger, and the driving effect of temperature on water is obvious, while the temperature difference at locations D, E, and F is smaller, the driving effect of temperature on water is not obvious, and the permeability of the bentonite sample with higher dry density is lower. In the experiment after the simulated water inflow, that is, when the hydraulic boundary is set at the left end, due to location F being closest to the hydraulic boundary at the left end, the volumetric water content at location F increases from the initial 0.237 to about 0.359 after 91.25 d and remains basically constant, which indicates that location F tends to be saturated. At the same time, the volume water content at locations E, D, C, and B near and far from the hydraulic boundary at the left end also increased significantly. The volumetric water content at location A, which is the farthest from the left hydraulic boundary, is gradually decreased with time (Figure 6b). This is mainly because the permeability of the bentonite sample with higher dry density is very small, and the deionized water injected from the hydraulic boundary at the left end cannot penetrate to location A. Moreover, location A is close to the high-temperature boundary at the right end, and the internal water migrates along the soil pores in the direction of the lower temperature under the action of the temperature gradient. Therefore, the volumetric water content at location A gradually decreases with time in a short period of time. At this time, the driving effect of temperature on water is obvious.

### 4.3. Thermal Conductivity at the Joints

According to the above experimental results, the thermal conductivity at the joint of the composite bentonite samples can be further calculated. The calculation equation of thermal conductivity is as follows:

(1) $$\lambda =\frac{Qd}{At\Delta T}$$

where *λ* is the thermal conductivity (W/(m∙K)) and *d* is the width between the joints of the composite bentonite sample (m), which, in this paper, refers to the distance between location B and those of C, D, and E, which is 0.03 m. *A* is the cross-sectional area of the soil sample (m^2^), and *t* is the time (s); from Figure 3, the temperature at different locations basically reached stability at 0.0125 d, so *t* = 1080 s. Δ*T* is the temperature difference between location B and those of C, D, and E (K), and *Q* is the heat energy (W∙s), which can be expressed as follows:

(2) Q=cmΔT

where *c* is the specific heat of the bentonite sample (J/(kg∙K)) and *m* is the mass (kg). According to [19], the relationship between specific heat (*c*) and mass water content (*w*) is

(3) $$c=k_{1}w+k_{2}$$

where *k*_1_ and *k*_2_ are the fitting parameters.

(4) m=ρV=ρAd

where *ρ* is the density of the bentonite sample (kg/m^3^), and the relationship with dry density (*ρ_d_*) and mass water content (*w*) is

(5) $$\rho =\rho_{d}(1+w)$$

(6) $$w=\theta \frac{\rho_{l}}{\rho_{d}}$$

where *θ* is the volumetric water content and *ρ_l_* is the density of the water (kg/m^3^), with *ρ_l_* = 1000 kg/m^3^.

By substituting Equations (2)–(6) into (1), we have

(7) $$\lambda =\frac{(k_{1}\theta \frac{\rho_{l}}{\rho_{d}}+k_{2})(\rho_{d}+\theta \rho_{l})d^{2}}{t}$$

Therefore, according to Equation (7), the thermal conductivity of the BC joint and DE joint of composite bentonite samples before and after simulated water inflow can be calculated as shown in Table 4. By comparing the thermal conductivity of the same joint location of the composite bentonite samples in the two cases in Table 4, it can be seen that compared with the experiment before simulated water inflow, after the hydraulic boundary is loaded at the left end, that is, the experiment after simulated water inflow, the volume water content at the joint location of the composite bentonite samples increases significantly at the end of the experiment and the thermal conductivity at the corresponding joint location also increases accordingly, which is the same as the previous conclusions [19,20,21,22,23]. As shown in Table 4, the thermal conductivity of the joint location after healing of the composite bentonite samples can meet the requirement of more than 0.8 W/(m∙K) proposed by the International Atomic Energy Agency (IAEA) [19,20,24], which can meet the thermal conductivity requirements of the engineering barrier of the repository.

### 4.4. Hydraulic Conductivity at the Joints

In the experiment after simulated water inflow, the composite bentonite samples were subjected to constant pressure water injection through the left hydraulic boundary. Therefore, the constant head method can be used to calculate the hydraulic conductivity (*K*) at the joint location of the composite bentonite samples:

(8) $$K=\frac{qd}{A\Delta Ht}$$

where *K* is the hydraulic conductivity (m/s), *q* is the amount of water seepage in time *t* (m^3^), *t* is the experimental time (s), and Δ*H* is the water level difference (m).

Therefore, according to Equation (8), the hydraulic conductivity of the BC joint and DE joint of the composite bentonite samples after simulated water inflow can be calculated as shown in Table 5. According to Table 5, it can be seen that the hydraulic conductivity of the joint location after healing of the composite bentonite samples is roughly the same as the hydraulic conductivity of the complete sample measured by the predecessors [24,25,26,27], which meets the low permeability requirements of the engineering barrier of the HLW repository being less than 10^−11^ m/s [24,25].

### 4.5. Healing of Joints

**Figure 7:**
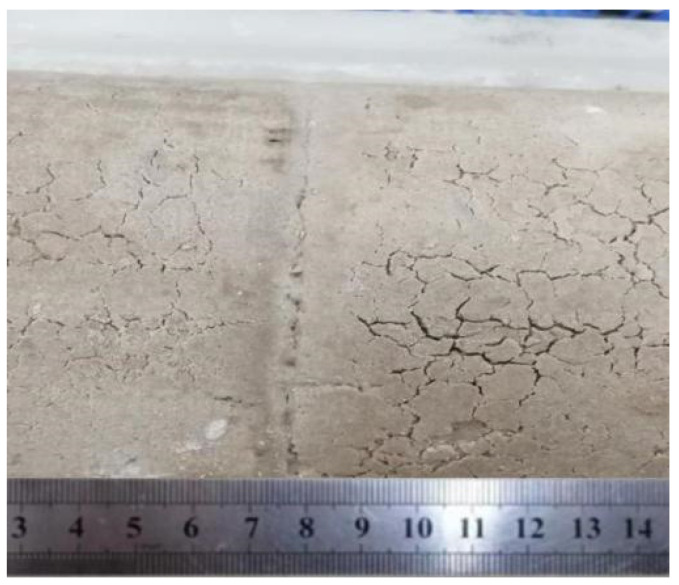
Healing at the DE joint of the composite bentonite samples.

Figure 7 shows a photo of the healing effect at the DE joint of the composite bentonite samples taken after the experiment. It can be seen that the healing effect of the DE joint area of the composite bentonite samples is good. Hydration of the bentonite composite samples produces volume swelling. Under the action of a swelling force, the structural plane between the joint and the sample basically disappears. Before the experiment, the initial dry density of the bentonite sample was 1.58 g/cm^3^. After the experiment, the dry density measured by sampling near the joint was 1.52 g/cm^3^. That is, the dry density of the bentonite sample near the joint gradually decreased. That is to say, the process of hydration swelling of the composite bentonite sample is accompanied by the adjustment of stress. The composite bentonite samples are continuously squeezed to the joint area after hydration swelling, the whole composite samples is generally homogenized [28,29,30], and the joints between the composite bentonite samples tend to heal.

## 5. Conclusions

In this paper, the laboratory experiments of heat conduction and water migration characteristics of composite bentonite samples before and after simulated water inflow were carried out by the developed laboratory experimental device. The evolution of temperature and volumetric water content at different locations of the composite bentonite samples with time in the two cases was obtained. After comparing and analyzing the experimental results in these two cases, the main conclusions are as follows:

(1) The variation trend of temperature at different locations of the composite bentonite samples with time is basically similar in the two cases. The loading hydraulic boundary condition makes the temperature near the hydraulic boundary increase slightly in the later stage of the experiment, but it has little effect on the temperature at other locations, which indicates that the change in internal temperature of the composite bentonite samples is mainly affected by the temperature boundary and that the change in the internal water has little effect on it.
(2) Due to the low permeability of the high-pressure compacted bentonite sample, in a short period of time, the loading of hydraulic boundary conditions only makes the volumetric water content of composite bentonite samples near the hydraulic boundary increase significantly but has little effect on other locations. Under the influence of temperature boundary, the internal water near the temperature boundary migrates along the soil pores in the direction of the hydraulic boundary under the action of a temperature gradient, so the volumetric water content gradually decreases with time in a short period of time.
(3) Based on the experimental results, the thermal conductivity and hydraulic conductivity of the joint location after healing of the composite bentonite samples were calculated. The calculation results show that the thermal conductivity and hydraulic conductivity of the joint location after healing can meet the thermal conductivity and low permeability requirements of the HLW repository engineering barrier.
(4) The hydration of the bentonite composite samples produces volume swelling. Under the action of the swelling force, the structural plane between the joint and the sample basically disappears. The process of hydration swelling of the composite bentonite sample is accompanied by the adjustment of stress, the composite bentonite samples are continuously squeezed to the joint area after hydration swelling, the whole composite samples is generally homogenized, and the joints between the composite bentonite samples tend to heal.

## Figures and Tables

**Table 1:** Mineral composition of GMZ bentonite.

Mineral	Quality Percentage/%
Montmorillonite	74.4
Quartz	12.4
Cristobalite	7.8
Feldspar	4.3
Calcite	0.4
Kaolinite	0.7

**Table 2:** Basic physical property parameters of GMZ bentonite.

Parameter	Value
Particle size/μm	<2
Liquid limit/%	170
Plasticity limit/%	27.43
Plasticity index	142.57
Specific gravity/(mg/m^3^)	2.66

**Table 3:** Initial temperature and final temperature at different locations in two cases.

Location	Experimental Case	Initial Temperature/°C	Final Temperature/°C	Δ*T*_1_/°C	Δ*T*_2_/°C
A	Before simulated water inflow	25.3	68.0	0.5	0.1
	After simulated water inflow	24.8	68.1		
B	Before simulated water inflow	25.2	41.4	0.5	0.2
	After simulated water inflow	24.7	41.6		
C	Before simulated water inflow	25.4	36.8	0.7	0.3
	After simulated water inflow	24.7	37.1		
D	Before simulated water inflow	25.5	32.8	1.0	0.3
	After simulated water inflow	24.5	33.1		
E	Before simulated water inflow	25.3	32.1	0.9	0.5
	After simulated water inflow	24.4	32.6		
F	Before simulated water inflow	25.6	31.1	1.4	0.9
	After simulated water inflow	24.2	32.0		

**Table 4:** Thermal conductivity at the joint in two cases.

Experimental Case		*k*_1_	*k*_2_	Δ*T*/K	*λ*/(W/(m·K))
Before simulated water inflow	BC joint	3695.2	198.9	277.95	1.173
	DE joint			273.75	1.159
After simulated water inflow	BC joint			276.95	1.236
0.269	DE joint			273.85	1.305

**Table 5:** Hydraulic conductivity at the joint after simulated water inflow.

Experimental Case		*t*/s	Δ*H*/m	*q*/m^3^	*K*/(m/s)
After simulated water inflow	BC joint	7.884 × 10^6^	2.039	4.477× 10^−6^	1.084 × 10^−12^
	DE joint			8.718 × 10^−6^	2.112 × 10^−12^

## Data Availability

The original contributions presented in the study are included in the article; further inquiries can be directed to the corresponding author.
